# Formulas to Explain Popular Oscillometric Blood Pressure Estimation Algorithms

**DOI:** 10.3389/fphys.2019.01415

**Published:** 2019-11-21

**Authors:** Anand Chandrasekhar, Mohammad Yavarimanesh, Jin-Oh Hahn, Shih-Hsien Sung, Chen-Huan Chen, Hao-Min Cheng, Ramakrishna Mukkamala

**Affiliations:** ^1^Department of Electrical and Computer Engineering, Michigan State University, East Lansing, MI, United States; ^2^Department of Mechanical Engineering, University of Maryland, College Park, MD, United States; ^3^Department of Medicine, National Yang-Ming University, Taipei, Taiwan

**Keywords:** arterial compliance, blood pressure measurement, cuff device, cuff-less device, derivative oscillometry, fixed ratios, mathematical model, oscillometry

## Abstract

Oscillometry is the blood pressure (BP) measurement principle of most automatic cuff devices. The oscillogram (which is approximately the blood volume oscillation amplitude-external pressure function) is measured, and BP is then estimated via an empirical algorithm. The objective was to establish formulas to explain three popular empirical algorithms in the literature—the maximum amplitude, derivative, and fixed ratio algorithms. A mathematical model of the oscillogram was developed and analyzed to derive parametric formulas for explaining each algorithm. Exemplary parameter values were obtained by fitting the model to measured oscillograms. The model and formulas were validated by showing that their predictions correspond to measurements. The formula for the maximum amplitude algorithm indicates that it yields a weighted average of systolic and diastolic BP (0.45 and 0.55 weighting) instead of commonly assumed mean BP. The formulas for the derivative algorithm indicate that it can accurately estimate systolic and diastolic BP (<1.5 mmHg error), if oscillogram measurement noise can be obviated. The formulas for the fixed ratio algorithm indicate that it can yield inaccurate BP estimates, because the ratios change substantially (over a 0.5–0.6 range) with arterial compliance and pulse pressure and error in the assumed ratio translates to BP error via large amplification (>40). The established formulas allow for easy and complete interpretation of perhaps the three most popular oscillometric BP estimation algorithms in the literature while providing new insights. The model and formulas may also be of some value toward improving the accuracy of automatic cuff BP measurement devices.

## Introduction

**Figure 1:**
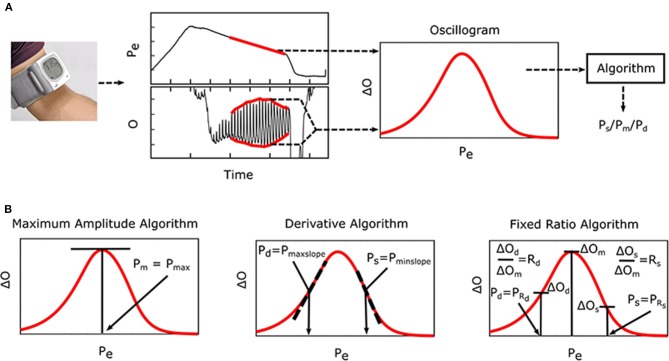
Oscillometric blood pressure (BP) measurement principle, which is employed by most automatic cuff devices, and associated algorithms. **(A)** The external pressure (*P*_*e*_) of an artery is swept via cuff inflation/deflation, and *P*_*e*_ (i.e., cuff pressure) is measured and high-pass filtered to yield oscillations (*O*). Systolic BP (*P*_*s*_), mean BP (*P*_*m*_), and/or diastolic BP (*P*_*d*_) are then estimated from the oscillogram [peak-to-peak amplitude or envelope difference of the oscillation (*O*) vs. *P*_*e*_ function] via an empirical algorithm. **(B)** Popular algorithms include the maximum amplitude, derivative, and fixed ratio algorithms (Ng and Small, 1994). Symbols of the features detected by each algorithm are defined as illustrated (see Glossary for text description).

Oscillometry is the blood pressure (BP) measurement methodology of most automatic cuff devices and can potentially be extended to achieve cuff-less and calibration-free monitoring of BP via widely used smartphones (Chandrasekhar et al., 2018a,b). Figure 1A illustrates the oscillometric BP measurement principle. The external pressure of an artery is swept between supra-systolic and sub-diastolic BP levels, and the external pressure is measured and high-pass filtered to yield oscillations indicative of the blood volume. Since the arterial compliance is dependent on transmural pressure (= BP—external pressure), the peak-to-peak amplitude of the blood volume

oscillations varies with the external pressure. BP is then estimated from the oscillation amplitude vs. external pressure function (i.e., “oscillogram”) via an algorithm. Figure 1B shows popular oscillometric BP estimation algorithms in the literature. The maximum amplitude algorithm estimates mean BP (i.e., the time average of instantaneous BP over the cardiac cycle) as the external pressure at which the oscillogram has peak value (Mauck et al., 1980; Drzewiecki et al., 1994). The fixed ratio algorithm estimates each of diastolic BP and systolic BP as the external pressure at which the oscillogram is some population-based fraction of its peak value (Geddes et al., 1982; Drzewiecki et al., 1994). The derivative algorithm estimates diastolic BP and systolic BP as the external pressures at which the oscillogram has maximum and minimum slope, respectively (Drzewiecki and Bronzino, 2006; Forouzanfar et al., 2015). Note that these algorithms are believed to be related to commercial device algorithms, which are proprietary (Van Montfrans, 2001; National High Blood Pressure Education Program/National Heart, 2002; Alpert et al., 2014).

The three algorithms are empirically-inspired rather than theoretically-based. In other words, they may have been conceived with the aid of reference BP measurements rather than first principles. Hence, it is difficult to understand their capabilities and limitations in estimating BP. As a result, the algorithms have previously been examined via mathematical modeling of oscillometry. More specifically, sensitivity analyses were applied to computational oscillometric models to determine the factors that affect the accuracy of the maximum amplitude algorithm (Ramsey, 1979; Ursino and Cristalli, 1996; Baker et al., 1997; Raamat et al., 1999) and fixed ratio algorithm (Drzewiecki et al., 1994; Ursino and Cristalli, 1996; Raamat et al., 2011; Liu et al., 2013). Here, we built upon the past modeling efforts by deriving parametric formulas to explain the popular oscillometric BP estimation algorithms and employing patient data to establish exemplary parameter values and to validate the formulas. The resulting closed-form expressions allow for easier and more complete interpretation of all three popular algorithms while providing new insights that are in contrast to some currently held beliefs about these algorithms.

## The Formulas

To derive formulas to explain the popular oscillometric BP estimation algorithms, we began with a previous mathematical model of the oscillogram, then extended this model, and finally formulated and solved the pertinent equations.

### Mathematical Model

**Figure 2:**
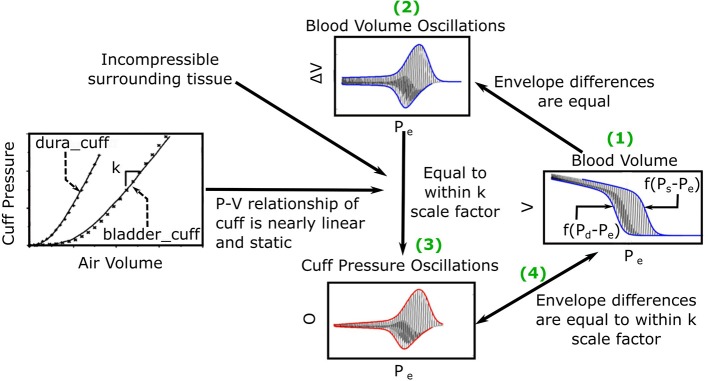
Previous mathematical model of the oscillogram (Liu et al., 2016, 2017). (1) The envelope difference of the unmeasured arterial blood volume (*V*) vs. external pressure (*P*_*e*_) function is equal to the difference in the x-axis reversed blood volume-transmural pressure relationships (*f*(*P* − *P*_*e*_)) evaluated at *P* = *P*_*s*_ and *P* = *P*_*d*_. (2) This envelope difference is equal to the envelope difference of the blood volume oscillation (*V*, i.e., high-pass filtered blood volume) vs. *P*_*e*_ function. (3) By assuming incompressible tissue around the artery and a linear and static cuff pressure-air volume relation, the latter envelope difference is proportional to the envelope difference of the measured oscillation (*O*) vs. *P*_*e*_ function (i.e., oscillogram) through a *k* (reciprocal of cuff compliance) scale factor. (The cuff-pressure-air volume relations shown are from two actual cuffs called dura_cuff and bladder_cuff.) (4) The oscillogram may thus be represented as *O* = *kf*(*P*_*s*_ − *P*_*e*_) − *kf*(*P*_*d*_ − *P*_*e*_).

The previous oscillogram model (Liu et al., 2016, 2017) is similar to other such models (Drzewiecki et al., 1994; Babbs, 2012) and is based on three major assumptions. First, the artery is purely elastic with a sigmoidal blood volume-transmural pressure relationship (*V* = *f*(*P*)). Second, the tissue around the artery is incompressible. Third, the cuff pressure-air volume relation is both static and linear such that the peak-to-peak amplitude of the measured oscillations (Δ*O*) is proportional to the peak-to-peak amplitude of the arterial blood volume oscillations (ΔV) via a constant *k*, which reflects the reciprocal of the compliance of the cuff. Figure 2 shows pictorially that these assumptions lead to the following model of the oscillogram:

(1) $$\begin{array}{l} \Delta O\;=kf\left(P_{s}-P_{e}\right)-kf\left(P_{d}-P_{e}\right), \end{array}$$

where *P*_*s*_ and *P*_*d*_ are systolic and diastolic BP, and *P*_*e*_ is the external pressure of the artery.

**Figure 3:**
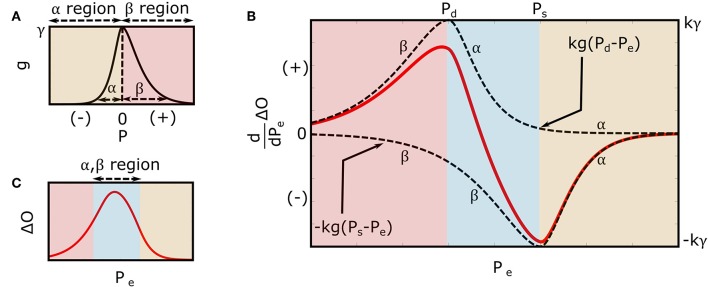
Extended mathematical model of the oscillogram. **(A)** Parametric model of the arterial compliance curve [$g=\frac{df}{dP_{e}}$ in Equation (3)] that is physiologic and readily leads to formulas for explaining the oscillometric BP estimation algorithms of Figure 1. The model parameters α and β reflect the compliance curve widths over negative and positive transmural pressures, respectively, while γ denotes the compliance curve height. **(B)** Model of the derivative of the oscillogram (*dO*/*dP*_*e*_ = *kg*(*P*_*d*_ − *P*_*e*_) − *kg*(*P*_*s*_ − *P*_*e*_)) obtained from the derivative of the model shown in Figure 2 and the arterial compliance curve model shown in **(A)**. **(C)** Model of the oscillogram obtained by integrating the derivative model shown in **(B)**.

We built upon this previous model by first differentiating Equation (1) with respect to *P*_*e*_ to yield the following model of the derivative of the oscillogram:

(2) $$\begin{array}{l} \frac{d\Delta O}{dP_{e}}=kg\left(P_{d}-P_{e}\right)-kg\left(P_{s}-P_{e}\right), \end{array}$$

where *g*(·) is the derivative of *f*(·) and represents the arterial compliance curve. We then conceived a parametric function for *g*(·) that fits experimental data, leads to closed-form expressions, and has a continuous, first derivative (to facilitate the derivation of some of the expressions) as follows:

(3) $$\begin{array}{l} g\left(P\right)=\gamma e^{\frac{P}{\alpha }}\left(-\frac{P}{\alpha }+1\right)u\left(-P\right)+\gamma e^{-\frac{P}{\beta }}\left(\frac{P}{\beta }+1\right)u\left(P\right), \end{array}$$

where *u*(·) is the unit-step function, and α, β, and γ are positive-valued parameters. As shown in Figure 3A, α and β reflect the arterial compliance curve widths over negative and positive transmural pressures, respectively, while γ denotes the height of the curve. Consistent with a sigmoidal blood volume-transmural pressure relation and experimental data (Drzewiecki et al., 1994), Equation (3) yields a skewed, unimodal arterial compliance curve that peaks near zero transmural pressure.

Substituting Equation (3) into Equation (2) yields the extended model of the derivative of the oscillogram as follows:

(4) $$\begin{array}{l} \frac{d\Delta O}{dP_{e}}=k\gamma e^{\frac{P_{d}-P_{e}}{\alpha }}\left(-\frac{P_{d}-P_{e}}{\alpha }+1\right)u\left(-\left(P_{d}-P_{e}\right)\right)\\ \;+k\gamma e^{-\frac{P_{d}-P_{e}}{\beta }}\left(\frac{P_{d}-P_{e}}{\beta }+1\right)u\left(P_{d}-P_{e}\right)\\ \;-k\gamma e^{\frac{P_{s}-P_{e}}{\alpha }}\left(-\frac{P_{s}-P_{e}}{\alpha }+1\right)u\left(-\left(P_{s}-P_{e}\right)\right)\\ \;-k\gamma e^{-\frac{P_{s}-P_{e}}{\beta }}\left(\frac{P_{s}-P_{e}}{\beta }+1\right)u\left(P_{s}-P_{e}\right). \end{array}$$

Integrating Equation (4) over *P*_*e*_ yields the extended model of the oscillogram as follows:

(5) $$\begin{array}{l} \Delta O=k\gamma \left(\left(P_{d}-P_{e}+2\beta \right)e^{-\frac{P_{d}-P_{e}}{\beta }}-\;\left(P_{s}-P_{e}+2\beta \right)e^{-\frac{P_{s}-P_{e}}{\beta }}\right)\\ \;u\left(P_{d}-P_{e}\right)+k\gamma (2\left(\alpha +\;\beta \right)+\;\left(P_{d}-P_{e}-2\alpha \right)e^{\frac{P_{d}-P_{e}}{\alpha }}\\ \;-\left(P_{s}-P_{e}+2\beta \right)e^{-\frac{P_{s}-P_{e}}{\beta }})\left(u(P_{e}-P_{d})-u(P_{e}-P_{s})\right)\\ \;+k\gamma \left(\left(P_{d}-P_{e}-2\alpha \right)e^{\frac{P_{d}-P_{e}}{\alpha }}-\;\left(P_{s}-P_{e}-2\alpha \right)e^{\frac{P_{s}-P_{e}}{\alpha }}\right)\\ \;u\left(P_{e}-P_{s}\right). \end{array}$$

Figure 3B illustrates the model predicted derivative of the oscillogram of Equation (4), while Figure 3C shows the model predicted oscillogram of Equation (5). These predictions qualitatively correspond to experimental data (see, e.g., Figure 1A). However, the extended model does carry a fourth assumption that the arterial compliance curve has a specific shape defined by Equation (3) with maximal value precisely at zero transmural pressure.

### Formula for the Maximum Amplitude Algorithm

As shown in Figure 1B, the maximum amplitude algorithm estimates mean BP (*P*_*m*_) as the external pressure at which the oscillogram has maximum value (*P*_max_). A formula for *P*_max_ may be found by setting Equation (4) to zero with _*P*_*e*_ = *P*max_ and invoking *P*_*d*_ < *P*_max_ < *P*_*s*_ (see Figure 3B) as follows:

(6) $$\begin{array}{l} \;k\gamma e^{\frac{\left(P_{d}-P_{\max}\right)}{\alpha }}\left(-\;\frac{P_{d}-P_{\max}}{\alpha }+1\right)\\ =k\gamma e^{-\frac{\left(P_{s}-P_{\max}\right)}{\beta }}\left(\;\frac{P_{s}-P_{\max}}{\beta }+1\right). \end{array}$$

The relevant solution to this equation is readily given as follows:

(7) $$\begin{array}{l} P_{\max}=\;\frac{\alpha }{\alpha +\;\beta }P_{s}+\frac{\;\beta }{\alpha +\;\beta }P_{d}. \end{array}$$

The final formula of Equation (7) indicates that the maximum amplitude algorithm yields a weighted average of systolic BP and diastolic BP where the weighting is determined by the arterial compliance curve widths.

### Formulas for the Derivative Algorithm

As shown in Figure 1B, the derivative algorithm estimates diastolic BP and systolic BP as the external pressures at which the oscillogram has maximum slope (*P*_*maxslope*_) and minimum slope (*P*_*minslope*_), respectively. A formula for *P*_*maxslope*_ may be found by employing Equation (4) with _*P*_*e*_ = *Pmaxslope*_, invoking *P*_*maxslope*_ < *P*_*d*_ (see Figure 3B), and taking the derivative and setting the equation to zero as follows:

(8) $$\begin{array}{l} \gamma e^{-\frac{\left(P_{d}-P_{maxslope}\right)}{\beta }}\left(\frac{P_{d}-P_{maxslope}}{\beta^{2}}\right)\\ =\;k\gamma e^{-\frac{\left(P_{s}-P_{maxslope}\right)}{\beta }}\left(\frac{P_{s}-P_{maxslope}}{\beta^{2}}\right). \end{array}$$

The solution to this equation is given as follows:

(9) $$\begin{array}{l} P_{maxslope}=P_{d}-\;\frac{PP}{e^{\frac{PP}{\beta }}-1}, \end{array}$$

where *PP* = *P*_*s*_ − *P*_*d*_ is the pulse pressure. Using a similar procedure but with *P*_*minslope*_ > *P*_*s*_ (see Figure 3B), the following formula for *P*_*minslope*_ results:

(10) $$\begin{array}{l} P_{minslope}=P_{s}+\;\frac{PP}{e^{\frac{PP}{\alpha }}-1}. \end{array}$$

The final formulas of Equations (9) and (10) indicate that the derivative algorithm underestimates diastolic BP and overestimates systolic BP by an amount that is dependent on both PP and the arterial compliance curve widths.

### Formulas for the Fixed Ratio Algorithm

As shown in Figure 1B, the fixed ratio algorithm estimates diastolic BP as the external pressure at which the rising portion of the oscillogram is some ratio of its maximal value (*P*_*R*_*d*__, where *R*_*d*_ is the assumed diastolic ratio) and systolic BP as the external pressure at which the falling portion of the oscillogram is some ratio of its maximal value (*P*_*R*_*s*__ where *R*_*s*_ is the assumed systolic ratios). Formulas for the true ratios [*TR*_*d*_ and *TR*_*s*_, i.e., the amplitude of the oscillogram at the actual systolic BP/diastolic BP (e.g., invasive BP values) divided by the maximal oscillogram amplitude] may be derived by substituting *P*_*s*_ or *P*_*d*_ and *P*_max_ for *P*_*e*_ into Equation (5) as follows:

(11) $$\begin{array}{l} TR_{d}=\frac{\Delta {O|}_{P_{e}=P_{d}}}{\Delta {O|}_{P_{e}=P_{\max}}}=\;\frac{2\beta -\left(PP\;+\;2\beta \right)e^{-\frac{PP}{\beta }}}{2\left(\alpha +\;\beta \right)-\left(PP+2\left(\alpha +\;\beta \right)\right)e^{-\frac{PP}{\alpha +\;\beta }}}. \end{array}$$

(12) $$\begin{array}{l} TR_{s}=\frac{\Delta {O|}_{P_{e}=P_{s}}}{\Delta {O|}_{P_{e}=P_{\max}}}=\;\frac{2\alpha -\left(PP\;+\;2\alpha \right)e^{-\frac{PP}{\alpha }}}{2\left(\alpha +\;\beta \right)-\left(PP+2\left(\alpha +\;\beta \right)\right)e^{-\frac{PP}{\alpha +\;\beta }}}. \end{array}$$

The final formulas of Equations (11) and (12) indicate that the true ratios vary with PP and the widths of the arterial compliance curve.

To derive formulas for indicating how much error in the presumptive systolic ratio (*R*_*s*_ − *TR*_*s*_) translates to error in systolic BP (*P*_*R*_*s*__ − *P*_*s*_), two cases must be considered. One case is an assumed ratio leading to systolic BP underestimation. In this case, the systolic ratio error may be determined from the middle term in Equation (5) along with Equation (12) as follows:

(13) $$\begin{array}{l} R_{s}-TR_{s}=\frac{\Delta {O|}_{P_{e}=P_{R_{s}}}}{\Delta {O|}_{P_{e}=P_{\max}}}-TR_{s}\\ \;=\;\frac{2\left(\alpha +\;\beta \right)+\;\left(P_{d}-P_{R_{s}}-2\alpha \right)e^{\frac{{P_{d}-P}_{R_{s}}}{\alpha }}-\;\left(P_{s}-P_{R_{s}}+2\beta \right)e^{-\frac{{P_{s}-P}_{R_{s}}}{\beta }}-2\alpha +\left(PP\;+\;2\alpha \right)e^{-\frac{PP}{\alpha }}\;}{2\left(\alpha +\;\beta \right)-\left(PP+2\left(\alpha +\;\beta \right)\right)e^{-\frac{PP}{\alpha +\;\beta }}}. \end{array}$$

By assuming small systolic BP error (*P*_*R*_*s*__ ≈ *P*_*s*_) and neglecting the terms $e^{\frac{{P_{d}-P}_{R_{s}}}{\alpha }}\approx e^{-\frac{PP}{\alpha }}$ (as justified in the Results section), Equation (13) may be linearized as follows:

(14) $$\begin{array}{l} R_{s}-TR_{s}\approx \;\frac{2\beta -\;\left(P_{s}-P_{R_{s}}+2\beta \right)\left(1-\;\frac{P_{s}-P_{R_{s}}}{\beta }\right)}{2\left(\alpha +\;\beta \right)-\left(PP+2\left(\alpha +\;\beta \right)\right)e^{-\frac{PP}{\alpha +\;\beta }}}\\ \;\approx \frac{P_{s}-P_{R_{s}}}{2\left(\alpha +\;\beta \right)-\left(PP+2\left(\alpha +\;\beta \right)\right)e^{-\frac{PP}{\alpha +\;\beta }}}. \end{array}$$

The other case is an assumed ratio leading to systolic BP overestimation. In this case, the systolic ratio error may be determined from the last term in Equation (5) along with Equation (12) as follows:

(15) $$\begin{array}{l} R_{s}-TR_{s}=\frac{\Delta {O|}_{P_{e}=P_{R_{s}}}}{\Delta {O|}_{P_{e}=P_{\max}}}-TR_{s}\\ \;=\frac{\left(P_{d}-P_{R_{s}}-2\alpha \right)e^{\frac{{P_{d}-P}_{R_{s}}}{\alpha }}-\;\left(P_{s}-P_{R_{s}}-2\alpha \right)e^{\frac{{P_{s}-P}_{R_{s}}}{\beta }}-2\alpha +\left(PP\;+\;2\alpha \right)e^{-\frac{PP}{\alpha }}\;}{2\left(\alpha +\;\beta \right)-\left(PP+2\left(\alpha +\;\beta \right)\right)e^{-\frac{PP}{\alpha +\;\beta }}}. \end{array}$$

By likewise simplifying Equation (15), the identical equation on the right-hand-side of Equation (14) results. Solving for *P*_*R*_*s*__ in this common equation thus yields the following small error formula:

(16) $$\begin{array}{l} P_{R_{s}}\;\approx P_{s}-\left(2\left(\alpha +\;\beta \right)-\left(PP+2\left(\alpha +\;\beta \right)\right)e^{-\frac{PP}{\alpha +\;\beta }}\right)\\ \;\left(R_{s}-TR_{s}\right). \end{array}$$

An analogous formula for translating diastolic ratio error to small diastolic BP error may be derived using a similar procedure but neglecting terms $e^{-\frac{{P_{s}-P}_{R_{d}}}{\beta }}\approx e^{-\frac{PP}{\beta }}$ and is given as follows:

(17) $$\begin{array}{l} P_{R_{d}}\;\approx P_{d}+\left(2\left(\alpha +\;\beta \right)-\left(PP+2\left(\alpha +\;\beta \right)\right)e^{-\frac{PP}{\alpha +\;\beta }}\right)\\ \;\left(R_{d}-{TR}_{d}\right). \end{array}$$

The final small error formulas of Equations (16) and (17) indicate that error in the presumptive ratios maps to error in BP by a scale factor determined by PP and the arterial compliance curve widths.

## Materials and Methods

To determine the parameter values and demonstrate the validity of the mathematical model and formulas, we analyzed patient data.

### Patient Data

We leveraged previously collected, de-identified patient data. These data are described in detail elsewhere (Cheng et al., 2012, 2013). Briefly, we started with data typically comprising two consecutive oscillometric cuff pressure waveforms via repeated inflation/deflation cycles of an upper arm cuff device (WatchBP Office, Microlife AG, Switzerland) and a reference brachial BP waveform via an intra-arterial catheter in the opposite arm from 33 cardiac catheterization patients before and after sublingual nitroglycerin administration. We excluded data based on three criteria: (1) invasive diastolic BP < the minimum Microlife device cuff pressure of ~60 mmHg or invasive systolic BP > the maximum Microlife device cuff pressure (to preclude inaccurate detection of the maximum and minimum slopes of the oscillogram); (2) obvious artifact in the oscillometric cuff pressure waveforms (which is not accounted for by the formulas) or unsteady brachial BP waveforms (to preclude unreliable reference measurements) as ascertained by visual inspection; or (3) inter-arm cuff BP differences > 10 mmHg (to likewise preclude unreliable reference measurements). A total of 28 baseline and 26 nitroglycerin measurement sets from 21 patients (age of 64 ± 14 years; 80% male; BMI of 27.2 ± 4.8 kg/m^2^; 24% diabetic; and 63% hypertensive) remained for analysis. The 54 total measurement sets comprised two measurement sets before nitroglycerin in 11 subjects and after nitroglycerin in eight subjects as well as one measurement set before nitroglycerin in six subjects and after nitroglycerin in ten subjects. Fourteen of the subjects had measurement sets both before and after nitroglycerin. The data notably covered a wide BP range (95–180 mmHg for reference systolic BP and 58–88 mmHg for reference diastolic BP).

### Data Analysis

We first constructed the oscillogram from the measured oscillometric cuff pressure waveforms. Our procedure was similar to that described elsewhere (Liu et al., 2016). Briefly, we (1) band-pass filtered the measured waveforms to extract the cuff pressure oscillations; (2) detected the maxima and minima of the oscillations; (3) filtered these extrema as a function of cuff pressure with a 10-mmHg rectangular window; (4) linearly interpolated the discrete data; and (4) subtracted the so-obtained upper and lower envelopes to yield the oscillogram.

We then analyzed the oscillograms to assess the validity of the model and to determine exemplary formula parameter values. In particular, we set *P*_*s*_ and *P*_*d*_ in Equation (5) to the average invasive systolic BP and diastolic BP during the time period of the oscillogram and set *k*γ in the equation so as to equate the peak values of the model predicted and measured oscillograms. We then estimated the two remaining free parameters, α and β, by (1) varying the parameters over a physiologic range (0 < α, β < 30); (2) computing the mean squared error between the model predicted oscillogram and the middle of the measured oscillogram (i.e., the oscillogram over the “foot-to-foot” cuff pressure range, wherein foot is defined analogously to the onset of a BP pulse) for each candidate pair of parameters; and (3) identifying the parameter pair that yielded the minimum mean squared error. We evaluated the model in terms of the root-mean-square of the fitting error between the model predicted and measured oscillograms normalized by the root-mean-square of the measured oscillogram as well as comparisons of the average α and β estimates using paired *t*-tests and expectations based on known physiology.

We finally analyzed the oscillograms to assess the validity of the formulas themselves. More specifically, we applied the popular algorithms to estimate BP from the oscillograms. To mitigate noise, especially when applying the derivative algorithm, we first fitted an asymmetric normal function similar to Equation (19) (see Discussion section), but with a non-zero mean value, to the middle of the oscillogram using the MATLAB fmincon function (interior-point algorithm). To establish the fixed ratio values, we computed the ratios at the invasive systolic BP and diastolic BP for each oscillogram and then averaged the ratios. We then assessed the formula predictions, with α and β set to their estimated values and *P*_*s*_ and *P*_*d*_ set to their invasive BP values, against the algorithm estimates in terms of a correlation plot and Bland-Altman plot (difference in the predicted and measured values vs. the accurate measured values rather than the average of the two values). Note that whenever repeated oscillometric cuff pressure waveforms were available, we averaged the pair of results.

## Results

We provide results to first demonstrate the validity of the mathematical model and provide exemplary parameter estimates and to then validate the formulas themselves.

### Mathematical Model and Parameter Estimates

**Figure 4:**
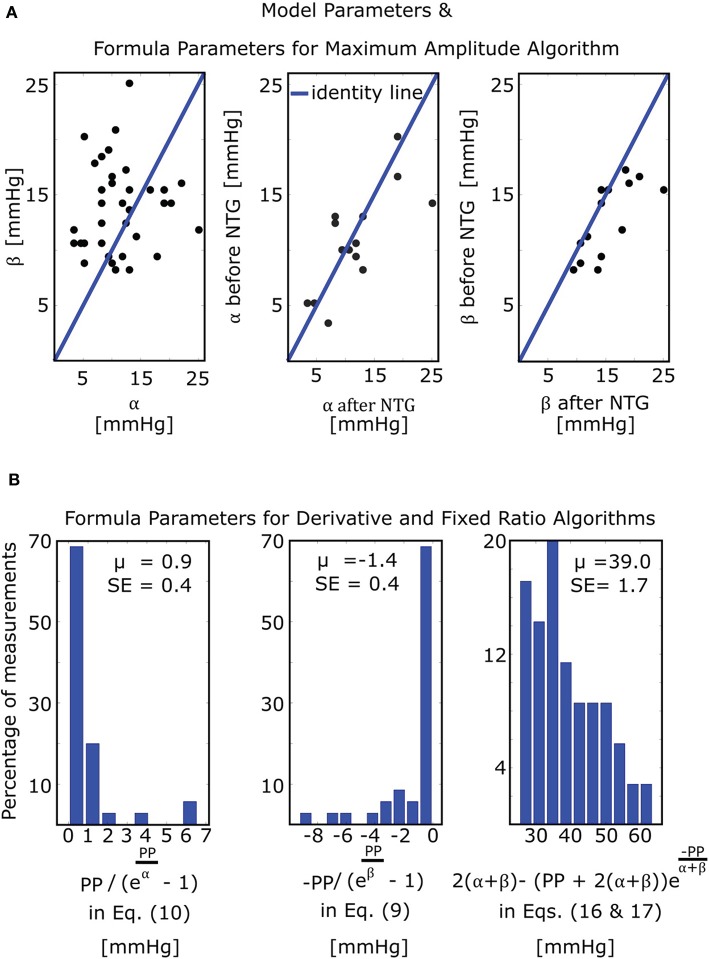
Model and formula parameter values obtained by fitting the model of Figure 3C to measured oscillograms from patients (M = 35 measurements before and/or after nitroglycerin (NTG) from N = 21 patients). **(A)** Model parameters and formula parameters for the maximum amplitude algorithm [see Equation (7)]. The parameters α and β again reflect the arterial compliance curve widths over negative and positive transmural pressures, respectively (see Figure 3A). **(B)** Formula parameters for the derivative and fixed ratio algorithms. The two histograms on the left show the values of the BP errors in the formulas for the derivative algorithm [see Equations (9) and (10)], while the histogram on the right shows the values of the scale factor mapping ratio error to small BP error in the formulas for the fixed ratio algorithm [see Equations (16) and (17)]. PP, pulse pressure; μ, mean value; SE, standard error.

The mathematical model of Equation (5) was able to fit the oscillograms with a normalized-root-mean-squared-error (NRMSE) of 8.5 ± 0.5% (mean ± SE). Figure 4A illustrates the resulting model parameter estimates. The β estimates were larger than the α estimates (13.8 ± 0.7 vs. 11.4 ± 0.9 mmHg; *p* = 0.03), which is consistent with the expected right-skewed compliance curve (Drzewiecki et al., 1994). The β estimates also increased after nitroglycerin administration (15.4 ± 1.2 vs. 12.8 ± 0.9 mmHg; *p* = 0.007), which is consistent with the expected drug-induced increase in arterial compliance over the physiologic positive transmural BP regime, while the α estimates did not change following the intervention (11.7 ± 1.6 vs. 10.8 ± 1.2 mmHg; *p* = NS).

### Formula for the Maximum Amplitude Algorithm

**Figure 5:**
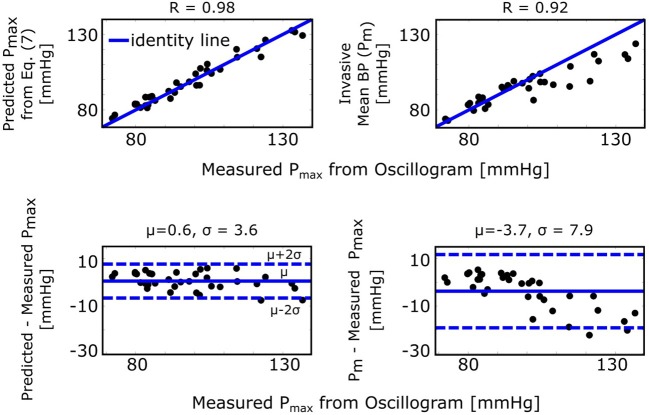
Validation results for the formula for explaining the maximum amplitude algorithm [see Figure 1 and Equation (7)]. Correlation and Bland-Altman plots of *P*_max_ (external pressure at which the oscillogram peaks) predicted by the formula vs. *P*_max_ measured from the oscillogram via the maximum amplitude algorithm (left plots) and of invasive mean BP (*P*_*m*_) vs. measured *P*_max_ (right plots). R is the correlation coefficient; μ, mean of the errors (bias error); and σ, standard deviation of the errors (precision error).

The maximum amplitude algorithm detects the external pressure at which the oscillogram peaks (*P*_max_), which has commonly been believed to denote mean BP. However, the formula of Equation (7) predicts that *P*_max_ is instead a weighted average of systolic BP and diastolic BP. Figure 5 (left) shows correlation and Bland-Altman plots of *P*_max_ predicted by the formula vs. *P*_max_ measured via the maximum amplitude algorithm. For comparison, Figure 5 (right) likewise shows invasive mean BP (*P*_*m*_) vs. measured *P*_max_. As can be seen, the formula predicted *P*_max_ well and clearly better than *P*_*m*_, especially at higher pressures.

### Formula for the Derivative Algorithm

**Figure 6:**
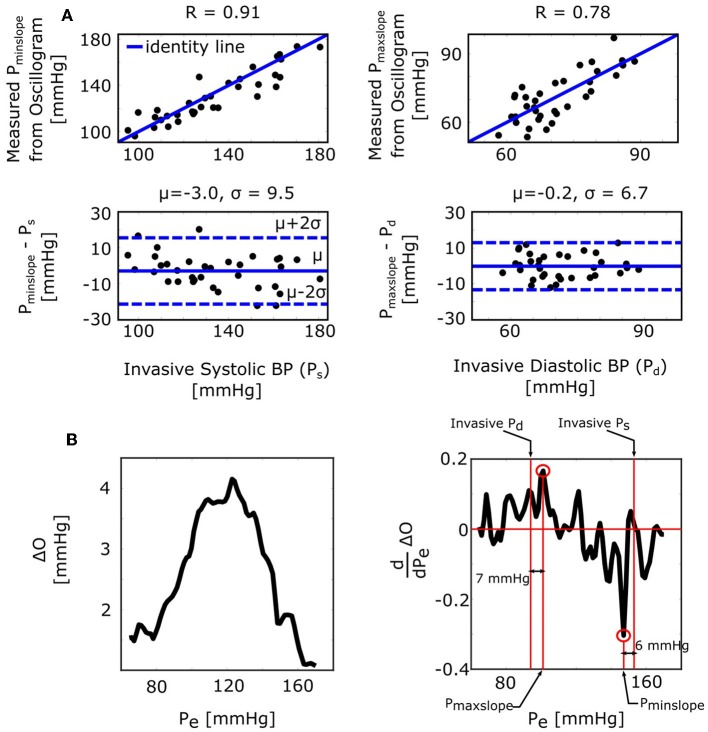
Validation results for the formulas for explaining the derivative algorithm [see Figure 1 and Equations (9) and (10)]. **(A)** Correlation and Bland-Altman plots of *P*_*maxslope*_ and *P*_*minslope*_ (external pressures at which the oscillogram has maximal and minimal slopes) measured from the oscillogram via the derivative algorithm vs. invasive diastolic BP (*P*_*d*_) and invasive systolic BP (*P*_*s*_). Consistent with the formula predictions, the bias errors are small. The appreciable precision errors are due to measurement noise, which is not considered by the formulas. **(B)** A representative oscillogram (before any filtering or model fitting) and its derivative illustrating the impact of typical high frequency measurement noise (due to, e.g., respiration, heart rate variability, and motion) on the correspondence between the detected *P*_*minslope*_/*P*_*maxslope*_ and *P*_*s*_/*P*_*d*_.

The derivative algorithm detects the external pressures at which the oscillogram has maximum slope (*P*_*maxslope*_) to estimate diastolic BP and minimum slope (*P*_*minslope*_) to estimate systolic BP. The formulas of Equations (9) and (10) predict that the BP errors of the derivative algorithm are $\frac{PP}{\left(e^{\frac{PP}{\beta }}-1\right)}$ for diastolic BP and $\frac{PP}{\left(e^{\frac{PP}{\alpha }}-1\right)}$ for systolic BP. Figure 4B shows histograms of the estimated $\frac{PP}{\left(e^{\frac{PP}{\beta }}-1\right)}$ and $\frac{PP}{\left(e^{\frac{PP}{\alpha }}-1\right)}$. Since these errors are small (0.9 ± 0.4 or − 1.4 ± 0.4 mmHg), the formulas predict that the derivative algorithm should yield accurate BP estimates. Figure 6A shows correlation and Bland-Altman plots of *P*_*maxslope*_ and *P*_*minslope*_ measured via the derivative algorithm vs. invasive diastolic BP (*P*_*d*_) and invasive systolic BP (*P*_*s*_). As can be seen, the bias errors (mean of the errors) are small, which is consistent with the formula predictions. However, the precision errors (standard deviation of the errors) are appreciable. The reason is surely due to derivative-induced amplification of oscillogram noise, which is common and often of high frequency character (due to, e.g., respiration, heart rate variability, and motion) but not considered by the formulas. Figure 6B illustrates a representative example of the impact of noise in the patient data on the derivative of the oscillogram before any filtering.

### Formulas for the Fixed Ratio Algorithm

**Figure 7:**
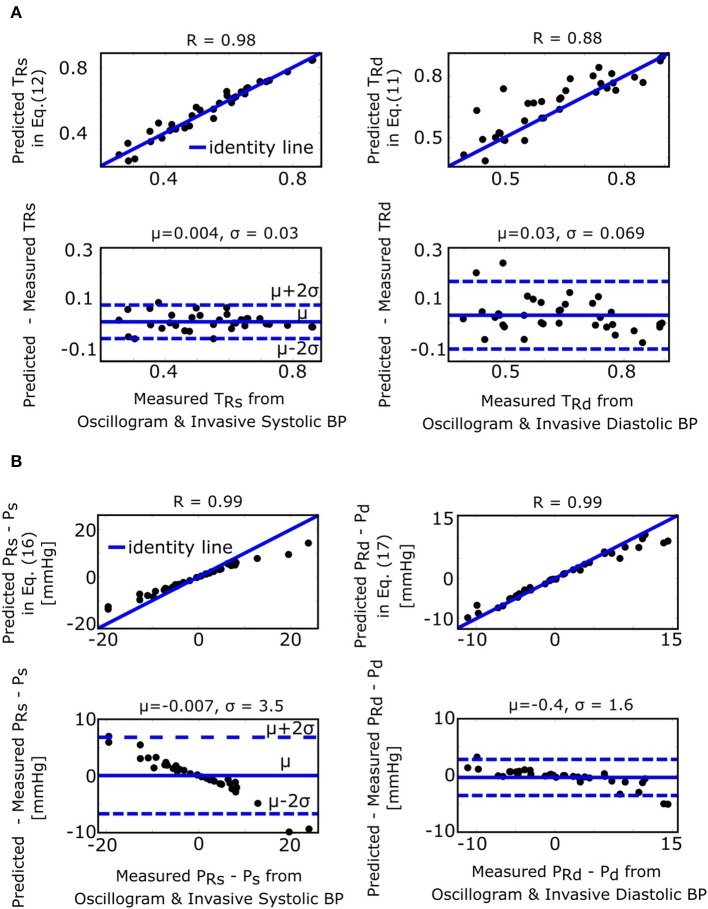
Validation results for the formulas for explaining the fixed ratio algorithm (see Figure 1). **(A)** Correlation and Bland-Altman plots of *TR*_*s*_ and *TR*_*d*_ (true systolic and diastolic ratios, i.e., the ratios of the oscillogram evaluated at the actual BP levels) predicted by the formulas of Equations (11) and (12) vs. *TR*_*s*_ and *TR*_*d*_ measured by evaluating the oscillogram at invasive systolic BP and diastolic BP. **(B)** Analogous plots of *P*_*R*_*s*__ − *P*_*s*_ and *P*_*R*_*d*__ − *P*_*d*_ (BP errors of fixed ratio algorithm) predicted by the small error formulas of Equations (16) and (17) vs. *P*_*R*_*s*__ − *P*_*s*_ and *P*_*R*_*d*__ − *P*_*d*_ measured as the difference between the BP estimates via application of the fixed ratio algorithm (with ratios given by the average of the measured *TR*_*s*_ and *TR*_*d*_ over all the patients) to the oscillogram and the invasive BP values.

The fixed ratio algorithm detects the external pressures at which the descending portion of the oscillogram is some assumed constant ratio of its maximal amplitude (*P*_*R*_*s*__) to estimate systolic BP and at which the ascending portion of the oscillogram is some assumed constant ratio of it maximal amplitude (*P*_*R*_*d*__) to estimate diastolic BP. The true systolic and diastolic ratios (*TR*_*s*_ and *TR*_*d*_) in the formulas of Equations (11) and (12) are defined as the ratios at which these externals pressures correspond to the actual BP levels. Figure 7A shows correlation and Bland-Altman plots of *TR*_*s*_ and *TR*_*d*_ predicted by the formulas of Equations (11) and (12) vs. *TR*_*s*_ and *TR*_*d*_ measured by evaluating the oscillogram at invasive systolic BP and diastolic BP. As can be seen, these formulas were generally able to predict the true ratios, which varied widely (over a 0.5–0.6 range). *P*_*R*_*s*__ − *P*_*s*_ and *P*_*R*_*d*__ − *P*_*d*_ in the formulas of Equations (16) and (17), respectively, represent the systolic BP and diastolic BP errors of the fixed ratio algorithm. Figure 7B shows analogous plots of *P*_*R*_*s*__ − *P*_*s*_ and *P*_*R*_*d*__ − *P*_*d*_ predicted by the small error formulas of Equations (16) and (17) vs. *P*_*R*_*s*__ − *P*_*s*_ and *P*_*R*_*d*__ − *P*_*d*_ measured as the difference between the BP estimates of the fixed ratio algorithm (with ratios given by the average of the measured *TR*_*s*_ and *TR*_*d*_ over all patients) and the invasive BP values. Note that these formulas neglected $e^{-\frac{PP}{\alpha }}$ and $e^{-\frac{PP}{\beta }}$, which is now justified by the small BP error terms of the formulas for the derivative algorithm (see Figure 4B). As can be seen, the formulas of Equations (16) and (17) were able to predict small BP errors but, as expected, became less accurate with increasing errors. Figure 4B additionally shows the histogram of the estimated $2\left(\alpha +\;\beta \right)-\left(PP+2\left(\alpha +\;\beta \right)\right)e^{-\frac{PP}{\alpha +\;\beta }}$ (the scale factor mapping ratio error to BP error) in the formulas of Equations (16) and (17). These estimates indicate that a ratio error of 0.2 (e.g., the assumed ratio is 0.5 but the true ratio is 0.7) would yield about an 8 mmHg BP error (39.0 ± 1.7 times 0.2).

## Discussion

This study is generally about mathematical modeling of the oscillometric BP measurement principle. While recent studies have employed such modeling toward improving oscillometric BP estimation accuracy (Babbs, 2012; Forouzanfar et al., 2012; Mukkamala et al., 2012; Liu et al., 2016, 2017), the purpose of this study was to establish parametric formulas with exemplary parameter values to explain three popular empirical algorithms in the literature for oscillometric BP estimation: (1) maximum amplitude, (2) derivative, and (3) fixed ratio algorithms (Figure 1). To derive the closed-form expressions, we extended a previous mathematical model of the oscillogram (Figures 2, 3) and then employed the extended model to formulate and solve the pertinent equations. To determine the formula parameter values, we fitted the model to oscillograms measured from patients covering a wide BP range (Figure 4).

A key step was to define a parametric function to represent the arterial compliance curve in the model (Equation 2) that is able to fit experimental data while leading to analytical solutions. We also sought a function that has a continuous, first derivative to readily arrive at the solutions. To satisfy these desired attributes, we conceived the function of Equation (3) (Figure 3A) and showed that the model with this function (Equation 5) can fit the measured oscillograms (8.5 ± 0.5% error). Other parametric functions to define the arterial compliance curve include an asymmetric exponential function (Baker et al., 1997; Babbs, 2012) and an asymmetric normal function as follows:

(18) $$\begin{array}{l} g_{\exp}\left(P\right)=\gamma_{\exp}e^{\frac{P}{\alpha_{\exp}}}u\left(-P\right)+\gamma_{\exp}e^{-\frac{P}{\beta_{\exp}}}u\left(P\right), \end{array}$$

(19) $$\begin{array}{l} g_{norm}\left(P\right)=\gamma_{norm}e^{-{\left(\frac{P}{\alpha_{norm}}\right)}^{2}}u\left(-P\right)+\gamma_{norm}e^{-{\left(\frac{P}{\beta_{norm}}\right)}^{2}}u\left(P\right). \end{array}$$

Equation (18) can lead to closed-form expressions. However, its first derivative is discontinuous, so the derivation is not as clean. More importantly, Equation (18) does not allow for better fitting of the measured oscillograms (9.3 ± 0.5% vs. 8.5 ± 0.5% error; *p* = 4.8 × 10^ − 4^ via paired *t*-test after log transformation of the data). While this quantitative difference in the fitting error may not seem large, the fitting difference can be seen visually through plots of model-predicted vs. measured oscillograms (see Supplemental Figure 1). Equation (19) does allow for better oscillogram fitting (7.7 ± 0.4% vs. 8.5 ± 0.5% error; *p* = 0.001). However, this equation does not lead to closed-form expressions, because, for example, the integral of a Gaussian cannot be solved analytically.

While we have not proven that Equation (3) is the optimal parametric arterial compliance curve function in terms of best data fitting while yielding closed-form expressions, we did demonstrate the validity of the resulting formulas by showing that they can predict experimental data (Figures 5–7). Note that these results also substantiate the secondary assumption of Equation (3) that the arterial compliance curve peaks at zero transmural pressure. For example, Figure 5 shows that the formula of Equation (7) predicts *P*_max_ detected by the maximum amplitude algorithm with little bias (0.6 mmHg). If the peak of the compliance curve were instead at an average of *Y* mmHg, then the bias would have been − *Y* mmHg.

**Figure 8:**
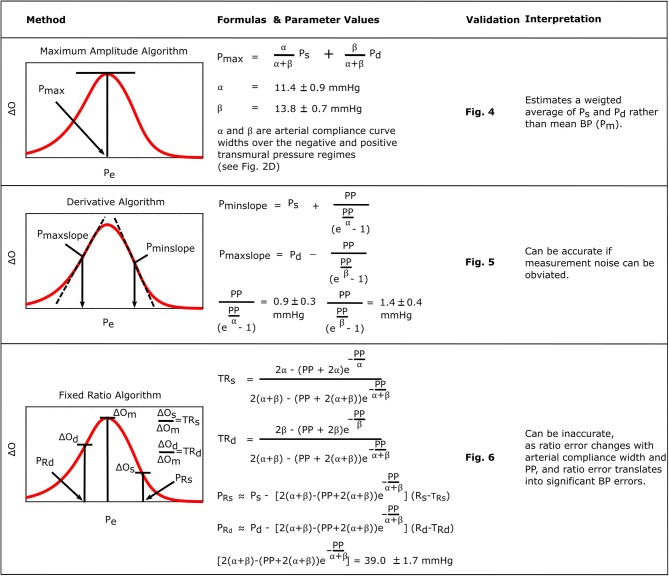
Summary of the established formulas for explaining popular oscillometric BP estimation algorithms.

Figure 8 consolidates and summarizes all of the established formulas. We interpret and discuss these formulas in the following.

The formula for the maximum amplitude algorithm indicates that the algorithm actually estimates a weighted average of systolic BP and diastolic BP (0.45 and 0.55 weighting here) in contrast to the commonly held belief that it yields an estimate of mean BP (compare left plots with right plots in Figure 5) (Mauck et al., 1980; Drzewiecki et al., 1994). An interesting coincidence is that a popular estimate of mean BP is obtained from systolic BP and diastolic BP as *P*_*m*_ = 0.4*P*_*s*_+0.6*P*_*d*_ (Bos et al., 2007). Since this estimate is imperfect and generally becomes less accurate with increasing pressure, it does not conflict with the new finding here that the maximum amplitude algorithm does not estimate mean BP. We also mention that, for this particular algorithm, the parametric arterial compliance curve functions of Equation (18) or (19) do lead to analytical formulas that likewise indicate that the algorithm yields a weighted average of systolic BP and diastolic BP instead of mean BP.

The formulas for the derivative algorithm (Figure 8) predict that the algorithm will overestimate systolic BP and underestimate diastolic BP but only by a small amount (<1.5 mmHg here), as PP is often substantially larger than the arterial compliance curve widths such that the two compliance curves in the model (Equation 2) are well-separated (Figure 3B). We also mention that the parametric arterial compliance curve of Equation (18) indicates that the derivative algorithm yields systolic BP and diastolic BP without any error (Babbs, 2012). This prediction is in contrast to a previous study indicating that the algorithm appreciably overestimates auscultation systolic BP (+9% bias error) and underestimates auscultation diastolic BP ( − 6% bias error) (Drzewiecki and Bronzino, 2006). These larger BP bias errors may be explained to a significant extent by the fact that auscultation underestimates systolic BP and overestimates diastolic BP (Noninvasive sphygmomanometers. Part 2: Clinical validation of automated measurement type., 2009). However, the formulas do not consider measurement noise. Since differentiation amplifies noise, common high frequency oscillogram measurement artifact due to, for example, respiration, heart rate variability, and motion is a major factor that can introduce appreciable BP precision errors in practice (Figure 6) (Babbs, 2012). Hence, the formulas suggest that if a robust algorithm for faithfully detecting the maximum and minimum slopes could be developed, the accuracy of oscillometric BP measurement could be significantly enhanced. The first two formulas for the fixed ratio algorithm (Figure 8) indicate that the true systolic and diastolic ratios vary with the arterial compliance curve widths and PP and thus considerably (0.5–0.6 in Figure 7A). This prediction is consistent with those of previous computational sensitivity analysis and modeling studies of the fixed ratio algorithm (Drzewiecki et al., 1994; Ursino and Cristalli, 1996; Rein Raamat et al., 2011; Babbs, 2012; Liu et al., 2013). However, these earlier studies only provided qualitative rather than exact relationships. The true ratio formulas here may be examined to glean further insight. By taking the derivative with respect to each of the three parameters (α, β, *PP*), it can be deduced that the numerator and denominator of the true ratio formulas (which are the same functions but with different parameter values) increase as each parameter increases and then plateau with further parameter increases. It can then be inferred that the true systolic ratio increases with increasing α and decreases with increasing β and *PP*, while the true diastolic ratio increases with increasing β and decreases with increasing α and *PP*. The last two formulas for the fixed ratio algorithm (Figure 8) indicate that error in the presumptive ratios translates to significant BP errors (e.g., a ratio error of 0.2 leads to an 8 mmHg BP error here). Note that the scale factor that maps ratio error to BP error is identical to the denominator of the true ratio formulas (Figure 8). Hence, the scale factor increases with α, β, or *PP*. Similarly, this prediction is consistent with and builds upon the previous computational sensitivity analysis studies (Drzewiecki et al., 1994; Ursino and Cristalli, 1996; Rein Raamat et al., 2011; Liu et al., 2013). However, these formulas are only valid for small errors, and larger ratio errors may be amplified even more to yield very large BP errors (Figure 7B). In sum, the fixed ratio algorithm may be generally inaccurate.

In conclusion, oscillometry is the BP measurement principle of most automatic cuff devices and has thus been a workhorse in hypertension management. This principle may also be emerging as a means for achieving cuff-less and calibration-free BP monitoring via smartphones (Chandrasekhar et al., 2018a,b) and may thus improve hypertension awareness and control rates. Oscillometric devices estimate BP from the measured oscillogram via an empirical algorithm. In this study, we explained perhaps the three most popular empirical algorithms in the literature (Ng and Small, 1994) through formulas. We specifically derived formulas based on a mathematical model of the oscillogram, determined exemplary formula parameter values by fitting the model to patient oscillograms, and validated the model and formulas using patient data. The resulting formulas are not merely confirmatory of present knowledge and past studies. In fact, the formula for the maximum amplitude algorithm indicates that the algorithm estimates a weighted average of systolic BP and diastolic BP rather than the commonly held belief that it estimates mean BP. Furthermore, the formulas for the derivative algorithm indicate that the algorithm can estimate systolic BP and diastolic BP with small bias errors, which is in contrast to a previous study indicating that it appreciably overestimates systolic BP and underestimates diastolic BP (Drzewiecki and Bronzino, 2006). The formulas for the fixed ratio algorithm add to previous modeling studies by indicating the precise dependency of the true ratios on arterial properties and the precise mapping of small ratio errors to small BP errors. In these ways, this study facilitates understanding of the capabilities and limitations of the important algorithms in estimating BP. The study may also be of some value toward improving algorithm accuracy, which has been called for in recent clinical publications (Picone et al., 2017; Muntner et al., 2019). For example, an optimization algorithm to fit the oscillogram model to the measured oscillogram [see (Babbs, 2012; Forouzanfar et al., 2012; Mukkamala et al., 2012; Liu et al., 2016, 2017)] may more accurately estimate BP. Alternatively, a simpler algorithm to faithfully identify the maximum and minimum oscillogram slopes in the presence of noise could allow the derivative algorithm to achieve low precision error.

## Data Availability Statement

The patient data in this manuscript can be made available upon reasonable request to C-HC (chench@vghtpe.gov.tw).

## Ethics Statement

Ethical review and approval was not required for the study on human participants in accordance with the local legislation and institutional requirements. The patients/participants provided their written informed consent to participate in this study.

## Author Contributions

AC developed the model and formulas, validated the model and formulas using patient data, and helped prepare the manuscript. MY helped develop the formulas and edited the manuscript. J-OH verified the model and formulas and edited the manuscript. S-HS, C-HC, and H-MC collected the patient data and edited the manuscript. RM guided the study and co-prepared the manuscript.

### Conflict of Interest

The authors declare that the research was conducted in the absence of any commercial or financial relationships that could be construed as a potential conflict of interest.

## Supplementary Material

Supplementary Figure 1 Examples of model-fitted oscillograms via different parametric arterial compliance curves (g(p)) versus measured oscillograms.

## Glossary of Symbols (in Order of Appearance in Figures)

*P*_*e*_: external pressure of an artery or measured cuff pressure

*O*: oscillations via high-pass filtering of *P*_*e*_

Δ*O*: peak-to-peak amplitude of *O*

Δ*O* vs. *P*_*e*_: oscillogram from which blood pressure (BP) is estimated via an algorithm

*P*_*s*_: systolic BP

*P*_*m*_: mean BP (i.e., time average of instantaneous BP over the cardiac cycle)

*P*_*d*_: diastolic BP

*P*_max_: *P*_*e*_ at which the oscillogram peaks, which is an estimate of *P*_*m*_ via the maximum amplitude algorithm

*P*_*maxslope*_: *P*_*e*_ at which the oscillogram has maximal slope, which is an estimate of *P*_*d*_ via the derivative algorithm

*P*_*minslope*_: *P*_*e*_ at which the oscillogram has minimal slope, which is an estimate of *P*_*s*_ via the derivative algorithm

*P*_*R*_*d*__: *P*_*e*_ at which the amplitude of the rising portion of the oscillogram is an assumed constant ratio of its maximal value (*R*_*d*_), which is an estimate of *P*_*d*_ via the fixed ratio algorithm

*P*_*R*_*s*__: *P*_*e*_ at which the amplitude of the falling portion of the oscillogram is an assumed constant ratio of its maximal value (*R*_*s*_), which is an estimate of *P*_*s*_ via the fixed ratio algorithm

*f*(·): function relating transmural pressure (*P* − *P*_*e*_) to blood volume (*V*), i.e., “arterial blood volume-transmural pressure relationship”

Δ*V*: peak-to-peak amplitude of *V*

*k*: reciprocal of the cuff compliance, i.e., scale factor that relates Δ*V* to Δ*O*

*g*(·) = *df*/*dP*: “arterial compliance curve”

α: parameter representing the width of the compliance curve over negative transmural pressure

β: parameter representing the width of the compliance curve over positive transmural pressure

γ: parameter denoting the height of the compliance curve

*T*_*R*_*s*__: amplitude of the falling portion of the oscillogram at the true *P*_*s*_ (e.g., invasive *P*_*s*_) divided by the maximal oscillogram amplitude, i.e., “true systolic ratio”

*T*_*R*_*d*__: amplitude of the rising portion of the oscillogram at the true *P*_*d*_ (e.g., invasive *P*_*d*_) divided by the maximal oscillogram amplitude, i.e., “true diastolic ratio”
