# Supplementary figures and images for: Formulas to Explain Popular Oscillometric Blood Pressure Estimation Algorithms
Source: Front Physiol. 2019 Nov 21;10:1415. doi: 10.3389/fphys.2019.01415 (PMC6881246; doi:10.3389/fphys.2019.01415)

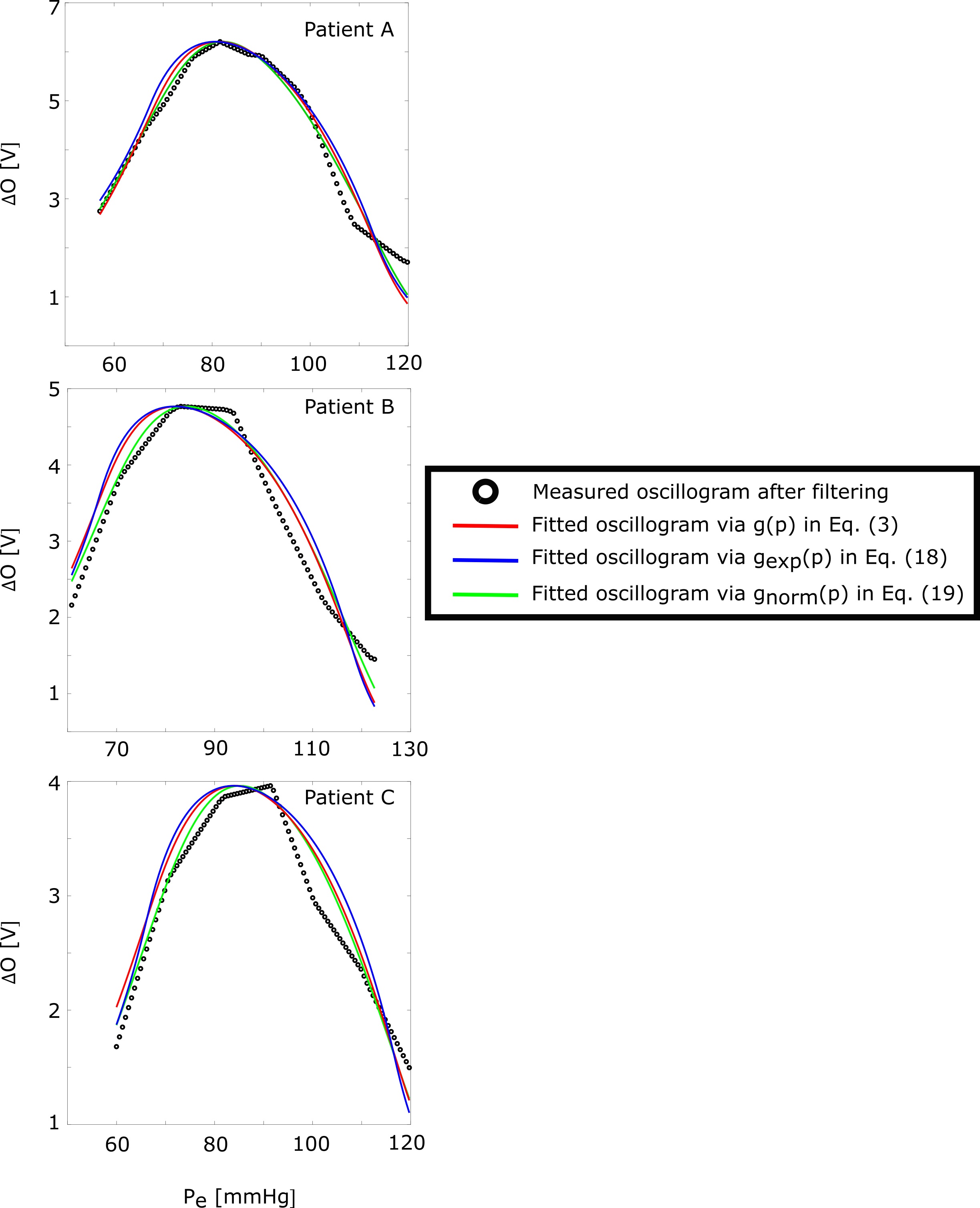

Supplement: Supplementary Figure 1 — Examples of model-fitted oscillograms via different parametric arterial compliance curves (g(p)) versus measured oscillograms. [file Image_1.JPEG]
